# Impact of whole-body and skeletal muscle composition on peak oxygen uptake in heart failure: a systematic review and meta-analysis

**DOI:** 10.1093/ehjopen/oeae082

**Published:** 2024-09-26

**Authors:** Veronika Schmid, Stephen J Foulkes, Devyn Walesiak, Jing Wang, Corey R Tomczak, Wesley J Tucker, Siddhartha S Angadi, Martin Halle, Mark J Haykowsky

**Affiliations:** Department for Preventive Sports Medicine and Sports Cardiology, TUM University Hospital, School of Medicine and Health, Technical University of Munich, Georg-Brauchle-Ring 56, 80992 Munich, Germany; Integrated Cardiovascular Exercise Physiology and Rehabilitation Lab, Faculty of Nursing, College of Health Science, University of Alberta, 3-045/11405 87 Ave NW, Edmonton, T6G IC9 Alberta, Canada; Integrated Cardiovascular Exercise Physiology and Rehabilitation Lab, Faculty of Nursing, College of Health Science, University of Alberta, 3-045/11405 87 Ave NW, Edmonton, T6G IC9 Alberta, Canada; Heart, Exercise and Research Trials Lab, St Vincent’s Institute of Medical Research, 9 Princes Street, Fitzroy, 3065 Victoria, Australia; Integrated Cardiovascular Exercise Physiology and Rehabilitation Lab, Faculty of Nursing, College of Health Science, University of Alberta, 3-045/11405 87 Ave NW, Edmonton, T6G IC9 Alberta, Canada; Division of Public Health, School of Medicine, University of Utah, 375 Chipeta Way, UT 84108 Salt Lake City, USA; College of Kinesiology, University of Saskatchewan, 87 Campus Dr, Saskatoon, S7N 5B2 Saskatchewan, Canada; Department of Nutrition and Food Sciences, Texas Woman’s University, 304 Administration Dr. Denton, TX 76204 Houston, USA; Department of Kinesiology, University of Virginia, 405 Emmet Street, VA 22903 Charlottesville, USA; Department for Preventive Sports Medicine and Sports Cardiology, TUM University Hospital, School of Medicine and Health, Technical University of Munich, Georg-Brauchle-Ring 56, 80992 Munich, Germany; DZHK (German Center for Cardiovascular Research), Partner Site Munich Heart Alliance, Munich, Bavaria, Germany; Integrated Cardiovascular Exercise Physiology and Rehabilitation Lab, Faculty of Nursing, College of Health Science, University of Alberta, 3-045/11405 87 Ave NW, Edmonton, T6G IC9 Alberta, Canada

**Keywords:** Heart failure, Peak oxygen uptake, Exercise intolerance, Body composition, Skeletal muscle

## Abstract

**Aims:**

Heart failure (HF) has a major impact on exercise tolerance that may (in part) be due to abnormalities in body and skeletal muscle composition. This systematic review and meta-analysis aims to assess how differences in whole-body and skeletal muscle composition between patients with HF and non-HF controls (CON) contribute to reduced peak oxygen uptake (VO_2_peak).

**Methods and results:**

The PubMed database was searched from 1975 to May 2024 for eligible studies. Cross-sectional studies with measures of VO_2_peak, body composition, or muscle biopsies in HF and CON were considered. Out of 709 articles, 27 studies were included in this analysis. Compared with CON, VO_2_peak [weighted mean difference (WMD): −9.96 mL/kg/min, 95% confidence interval (CI): −11.71 to −8.21), total body lean mass (WMD: −1.63 kg, 95% CI: −3.05 to −0.21), leg lean mass (WMD: −1.38 kg, 95% CI: −2.18 to −0.59), thigh skeletal muscle area (WMD: −10.88 cm^2^ , 95% CI: −21.40 to −0.37), Type I fibres (WMD: −7.76%, 95% CI: −14.81 to −0.71), and capillary-to-fibre ratio (WMD: −0.27, 95% CI: −0.50 to −0.03) were significantly lower in HF. Total body fat mass (WMD: 3.34 kg, 95% CI: 0.35–6.34), leg fat mass (WMD: 1.37 kg, 95% CI: 0.37–2.37), and Type IIx fibres (WMD: 7.72%, 95% CI: 1.52–13.91) were significantly higher in HF compared with CON. Absolute VO_2_peak was significantly associated with total body and leg lean mass, thigh skeletal muscle area, and capillary-to-fibre ratio.

**Conclusion:**

Individuals with HF display abnormalities in body and skeletal muscle composition including reduced lean mass, oxidative Type I fibres, and capillary-to-fibre ratio that negatively impact VO_2_peak.

## Graphical abstract

**Graphical Abstract:**
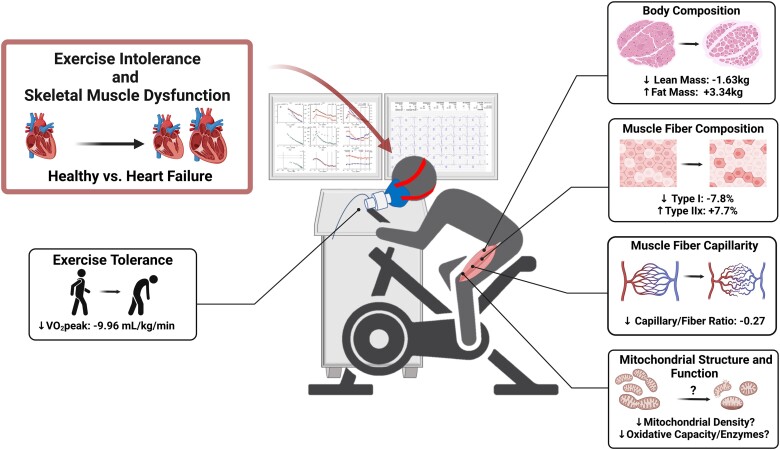

## Introduction

Decreased exercise tolerance, measured as reduced peak oxygen uptake (VO_2_peak), is a hallmark feature in heart failure (HF) and is associated with a reduced quality of life (QoL) and an increased risk of hospitalizations and mortality.^1,2^ The mechanisms underpinning the lower VO_2_peak in HF are multifactorial and traditionally have focused on impaired cardiac function.^3^ Non-cardiac ‘peripheral’ factors (i.e. reduced lean mass, oxidative fibres, and capillarity) may also contribute to reduced exercise tolerance in HF.^2^ However, due to inter-individual variability coupled with differences in physical activity levels between patients with HF and age-matched healthy controls, uncertainty remains regarding the magnitude of the decline in lean mass and skeletal muscle composition in HF and their contribution to the reduced VO_2_peak in HF. To address this knowledge gap, we performed a systematic review and meta-analysis to examine how differences in whole-body and skeletal muscle composition contribute to the lower VO_2_peak in HF compared with CON.

## Methods

### Data sources and search strategy

A PubMed literature search was conducted for English-language articles published between 1975 and May 2024. The search strategy was structured using the following three main terms: (i) heart failure; (ii) exercise tolerance, oxygen consumption, or VO_2_peak; and (iii) skeletal muscle and body composition or biopsy. In addition, a manual search was conducted using the reference list of the included studies in this review using Google Scholar. Studies identified from the database search were exported in full sets and transferred into the Covidence review management software (Melbourne, Australia). All studies were initially screened by title and abstract and subsequently by full text if they met the relevant inclusion criteria. Data from all included studies were extracted and reviewed by two independent researchers (V.S. and D.W.). In the context of studies with duplicate data, the study with the most relevant information was included to avoid overlapping populations. No ethical approval for this study was necessary as all data were sourced from previously published studies and did not involve any personally identifiable information.

### Study selection and inclusion and exclusion criteria

Only studies were included that: (i) compared subjects with HF [HF with reduced ejection fraction (HFrEF) or preserved ejection fraction (HFpEF)] with a non-HF control group (CON); (ii) reported VO_2_peak measured from expired gas analysis; and (iii) measured whole-body composition [measured by dual-energy X-ray absorptiometry (DEXA), magnetic resonance imaging (MRI), or computed tomography (CT)] and/or skeletal composition by muscle biopsy. The following criteria were excluded: (i) no original or duplicate data; (ii) no control group without HF; (iii) non-human cohorts (i.e. animal models); (iv) non-English studies; (v) no peak exercise data; and (vi) no body composition (by DEXA, MRI, or CT) and/or no skeletal muscle biopsy data.

### Study quality assessment

The quality of the included studies was evaluated by using the AXIS appraisal tool. The AXIS tool is a validated 20-point tool designed to assess the quality of cross-sectional studies (a maximum score of 20 indicates the highest quality).^4^

### Data synthesis and statistical analysis

Differences, measured as effect sizes, in primary and secondary outcomes between HF and CON group were quantified using a meta-analysis based on the random-effects models. From the models, the weighted average effect size, defined as the weighted mean difference (WMD) with 95% confidence intervals (CIs), was calculated for each outcome between HF and CON. The weight assigned to each study was the inverse of the variance within study. The larger the sample size and smaller the variance of the study, the greater the assigned weight. Heterogeneity within individual effect sizes was calculated by *I*^2^ and τ^2^. Forest plots were generated to illustrate individual effect sizes, standard deviations, and the associated *P*-value for hypothesis testing at an alpha level of 0.05. In addition, to determine the association between VO_2_peak and parameters of body and skeletal muscle composition, meta-regression analyses were performed using the R metacont package (R Core Team 2016, R Foundation for Statistical Computing, Vienna, Austria).

## Results

**Figure 1:**
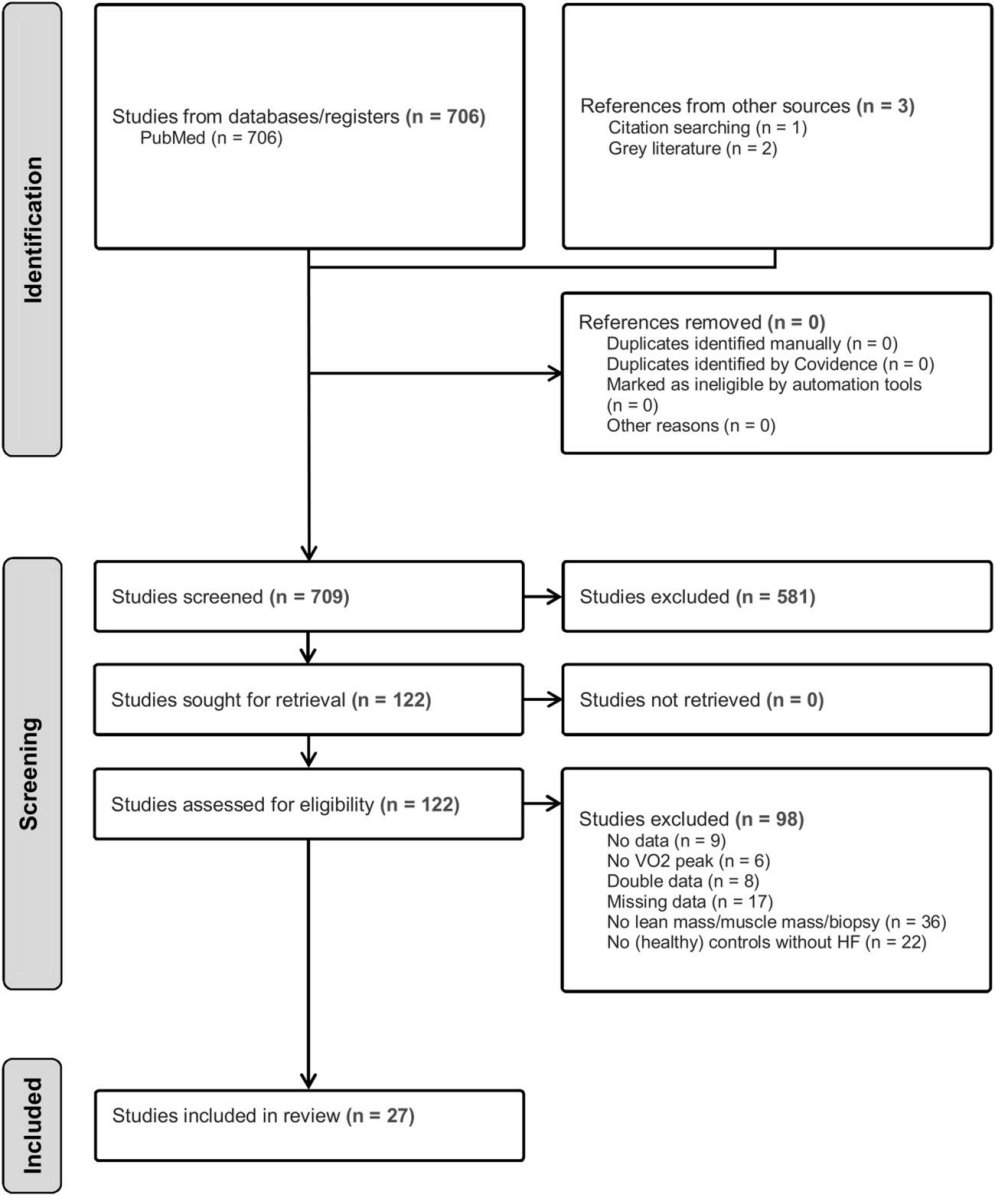
A PRISMA diagram for selection of studies included in the meta-analysis.

The initial search yielded 709 articles. Following screening, 27 studies that met the inclusion criteria were included in the analysis (*Figure 1*). Overall, 844 patients with HF (mean age: 64 years, 77% male, 67% HFrEF, and 33% HFpEF) and 515 CON participants (mean age: 62 years and 75% male) were included (*Table 1*). Cardiopulmonary exercise testing (CPET) was performed on a cycle ergometer (*n* = 15 studies) or treadmill (*n* = 12 studies) to quantify VO_2_peak. As detailed in *Table 1*, in addition to measuring VO_2_peak (*n* = 27 studies), 19 studies also measured body composition, whereas 13 studies measured skeletal muscle morphology. Body composition was assessed with either DEXA (*n* = 16 studies), MRI (*n* = 2 studies), or CT (*n* = 3 studies), while muscle fibre composition was evaluated with biopsy from the *m. vastus lateralis* (*n* = 13 studies).

**Table 1:** Demographic data

Author	Year	Population	Number/sex	Age	BW	BMI	CPET mode	Body composition technique	Skeletal muscle morphology & function
Sullivan^5^	1990	HFrEF CON	11 male 9 male	58 yrs. 49 yrs.	69 kg 76 kg	NR NR	CYC	N/A	*m. vastus lateralis* biopsy: Fibre composition, enzyme activity
Massie^6^	1996	HFrEF CON	18 male 8 male	64 yrs. 65 yrs.	NR NR	NR NR	CYC	N/A	*m. vastus lateralis* biopsy: Fibre composition
Harrington^7^	1997	HFrEF CON	100 male 31 male	59 yrs. 59 yrs.	NR NR	26.6 kg/m^2^ 26.3 kg/m^2^	TM	CT: Total muscle and quadriceps cross-sectional area	N/A
Toth^8^	1997	HFrEF CON	13 male/female 50 male, 2 female	72 yrs. 69 yrs.	NR NR	NR NR	TM	DEXA: Total body fat and lean mass, total and leg skeletal muscle mass	N/A
Mettauer^9^	2001	HFrEF Sedentary CON	13 male, 2 female 10 male, 1 female	53 yrs. 51 yrs.	73.3 kg 87.2 kg	NR NR	CYC	N/A	*m. vastus lateralis* biopsy: Fibre composition, enzyme activity, mitochondrial function
Duscha^10^	2002	HFrEF CON	25 male, 13 female 10 male, 11 female	54,50 yrs. 61,54 yrs.	NR NR	27.2 kg/m^2^ 27.3 kg/m^2^	CYC	N/A	*m. vastus lateralis* biopsy: Fibre composition, enzyme activity
Bekedam^11^	2003	HFrEF CON	5 male 5 male, 1 female	63 yrs. 51 yrs.	79.2 kg 81.3 kg	25.7 kg/m^2^ 25.4 kg/m^2^	CYC	N/A	*m. vastus lateralis* biopsy: Fibre composition, enzyme activity
Schulze^12^	2004	HFrEF CON	17 male 12 male	60 yrs. 59 yrs.	80.4 kg 87.8 kg	27.0 kg/m^2^ 26.8 kg/m^2^	CYC	CT: Thigh fat and muscle cross-sectional area	N/A
Williams^13^	2004	HFrEF CON	13 male, 1 female 7 male, 1 female	68 yrs. 63 yrs.	84 kg 78 kg	28.0 kg/m^2^ 26.0 kg/m^2^	CYC	DEXA: Total body lean mass, thigh lean mass	*m. vastus lateralis* biopsy: Fibre composition, enzyme activity
Toth^14^	2005	HFrEF CON	10 male 11 male	63 yrs. 70 yrs.	80 kg 80 kg	NR NR	TM	DEXA: Total body fat mass and lean mass, appendicular and leg muscle mass	*m. vastus lateralis* biopsy: Fibre composition
Piepoli^15^	2006	HFrEF CON	102 male, 5 female 22 male, 2 female	62 yrs. 59 yrs.	NR NR	26.2 kg/m^2^ 27.0 kg/m^2^	TM	DEXA: Total body fat and lean mass, leg fat and lean mass	N/A
Bekedam^16^	2009	HFrEF CON	14 male, 2 female 4 male, 1 female	64 yrs. 51 yrs.	79.0 kg 81.2 kg	26.5 kg/m^2^ 25.6 kg/m^2^	CYC	N/A	*m. vastus lateralis* biopsy: Fibre composition, enzyme activity
Miller^17^	2009	HFrEF/HFpEF CON	7 male, 3 female 6 male, 4 female	72 yrs. 69 yrs.	92.4 kg 81.9 kg	NR NR	TM	DEXA: Total body fat and lean mass, leg lean mass CT: Thigh cross-sectional area	*m. vastus lateralis* biopsy: Fibre composition, catabolic marker
Esposito^18^	2010	HFrEF CON	12 male 8 male	53 yrs. 52 yrs.	98 kg 88 kg	NR NR	CYC	N/A	*m. vastus lateralis* biopsy: Fibre composition, mitochondrial function
Toth^19^	2010	HFrEF/HFpEF CON	7 male, 4 female 7 male, 4 female	70 yrs. 70 yrs.	86.1 kg 85.6 kg	NR NR	TM	DEXA: Total body fat and lean mass, leg lean mass	N/A
Savage^20^	2011	HFrEF CON	7 male, 3 female 6 male, 5 female	73 yrs. 72 yrs.	95.6 kg 85.5 kg	NR NR	TM	DEXA: Total body fat and lean, leg lean mass, appendicular muscle mass	N/A
Haykowsky^21^	2013	HFpEF CON	19 male, 41 female 20 male, 20 female	70 yrs. 69 yrs.	81.1 kg 75.9 kg	29.9 kg/m^2^ 25.8 kg/m^2^	CYC	DEXA: Total body fat and lean mass, leg fat and lean mass	N/A
Zavin^22^	2013	HFrEF CON	31 male 39 male	67 yrs. 65 yrs.	86.0 kg 86.6 kg	NR NR	TM	DEXA: Total body fat and lean mass, leg fat and lean mass	N/A
Forman^23^	2014	HFrEF CON	24 male 30 male	67 yrs. 66 yrs.	88.3 kg 87.4 kg	29.4 kg/m^2^ 29.8 kg/m^2^	TM	DEXA: Total body fat and lean mass, leg fat and lean mass	*m. vastus lateralis* biopsy: Catabolic marker
Haykowsky^24^	2014	HFpEF CON	8 male, 15 female 4 male, 11 female	69 yrs. 70 yrs.	84 kg 67 kg	30.4 kg/m^2^ 24.6 kg/m^2^	CYC	MRI: Total thigh area subcutaneous fat, skeletal muscle and intermuscular fat of thigh	N/A
Kitzman^25^	2014	HFpEF CON	4 male, 18 female 21 male, 22 female	70 yrs. 69 yrs.	79.9 kg 78.0 kg	29.7 kg/m^2^ 26.7 kg/m^2^	CYC	N/A	*m. vastus lateralis* biopsy: Fibre composition
Panizzolo^26^	2015	HFrEF CON	7 male, 4 female 9 male, 6 female	62 yrs. 61 yrs.	72.8 kg 69.9 kg	25.6 kg/m^2^ 23.5 kg/m^2^	TM	DEXA: Total body lean mass	N/A
Keller-Ross^27^	2016	HFrEF CON	7 male, 2 female 7 male, 1 female	60 yrs. 63 yrs.	NR NR	31.9 kg/m^2^ 25.3 kg/m^2^	CYC	DEXA: Total fat and lean mass, leg fat and lean mass	N/A
Haykowsky^28^	2018	HFpEF CON	19 male, 81 female 23 male, 38 female	67 yrs. 69 yrs.	105.5 kg 74.5 kg	39.3 kg/m^2^ 25.9 kg/m^2^	TM	DEXA: Total body fat and lean mass MRI: Total thigh area, thigh skeletal muscle area and intramuscular fat	N/A
Munch^29^	2018	HFrEF CON	7 male, 1 female 5 male, 1 female	58 yrs. 66 yrs.	89 kg 77 kg	29.0 kg/m^2^ 25.0 kg/m^2^	CYC	DEXA: Total body fat and lean mass	N/A
Zamani^30^	2021	HFpEF CON	7 male, 13 female 14 male, 6 female	67 yrs. 54 yrs.	99.1 kg 81.4 kg	32.1 kg/m^2^ 26.7 kg/m^2^	CYC (supine)	DEXA: Total body lean mass, appendicular lean mass	*m. vastus lateralis* biopsy: Fibre composition, enzyme activity
Loncar^31^	2023	HFrEF/HFpEF CON	141 male 14 male	69 yrs. 68 yrs.	89.0 kg 78.0 kg	28.0 kg/m^2^ 25.0 kg/m^2^	TM	DEXA: Total body fat and lean mass, leg fat and lean mass	N/A

Data are presented as mean unless otherwise specified

CPET, cardiopulmonary exercise testing; CG, control group; CT, computed tomography; CYC, cycling; DEXA, dual-energy X-ray absorptiometry; HF, heart failure; HFpEF, heart failure with preserved ejection fraction; HFrEF, heart failure with reduced ejection fraction; MRI, magnetic resonance imaging; N/A, not available; TM, treadmill; yrs., years

### Peak oxygen uptake

**Figure 2:**
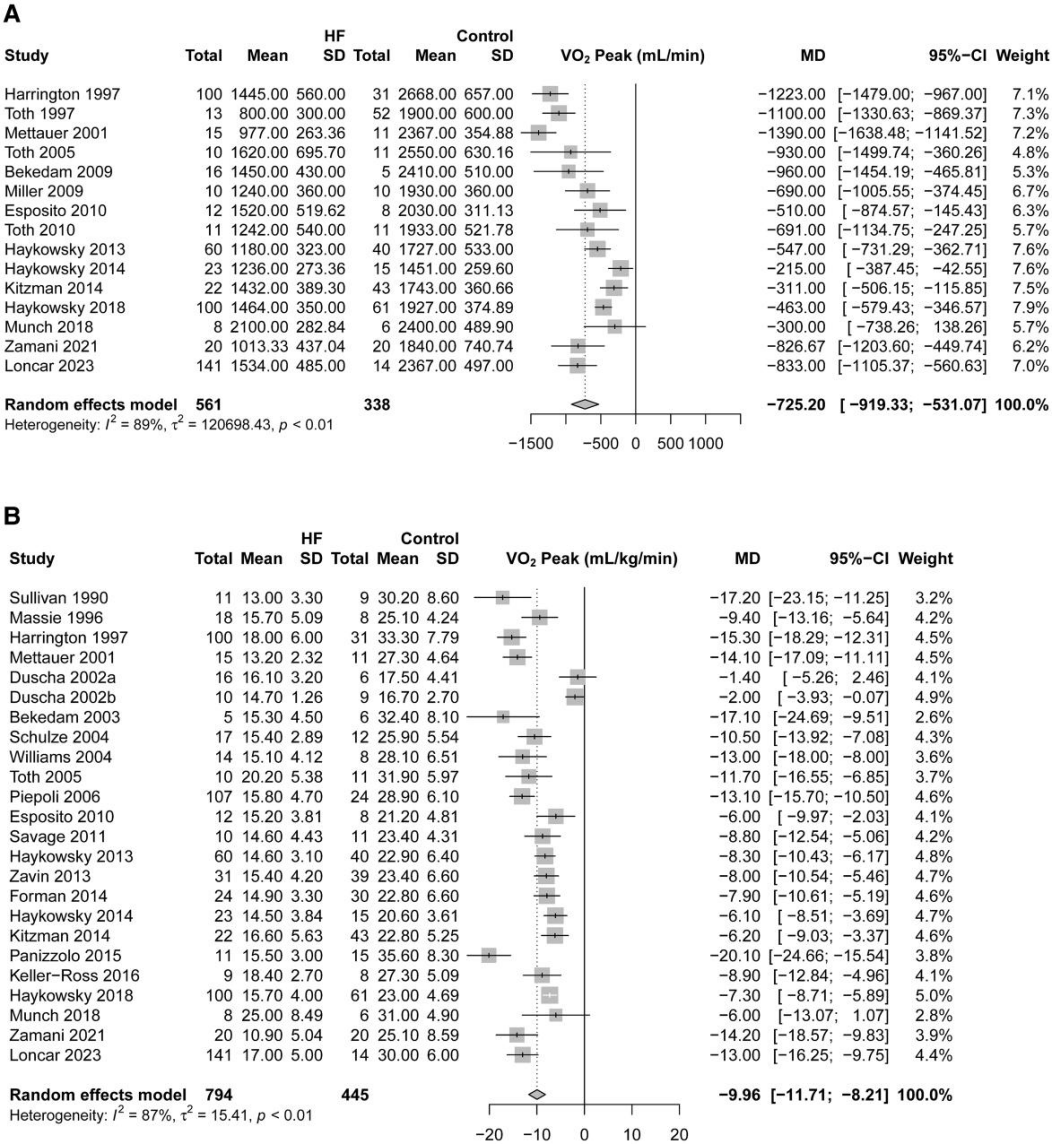
A peak oxygen uptake in absolute units (mL/min; *A*) and indexed to body weight (mL/kg/min; *B*) in patients with heart failure and controls.

Peak oxygen uptake was significantly and markedly lower in HF when measured in absolute values (WMD: −725.20 mL/min, 95% CI: −919.33 to −53.07 mL/min, *I*^2^ = 89%, *n* = 899) or indexed to whole-body mass (WMD: −9.96 mL/kg/min, 95% CI: −11.71 to −8.21 mL/kg/min, *I*^2^ = 87%, *n* = 1239) (*Figure 2*).

### Body composition outcomes

**Figure 3:**
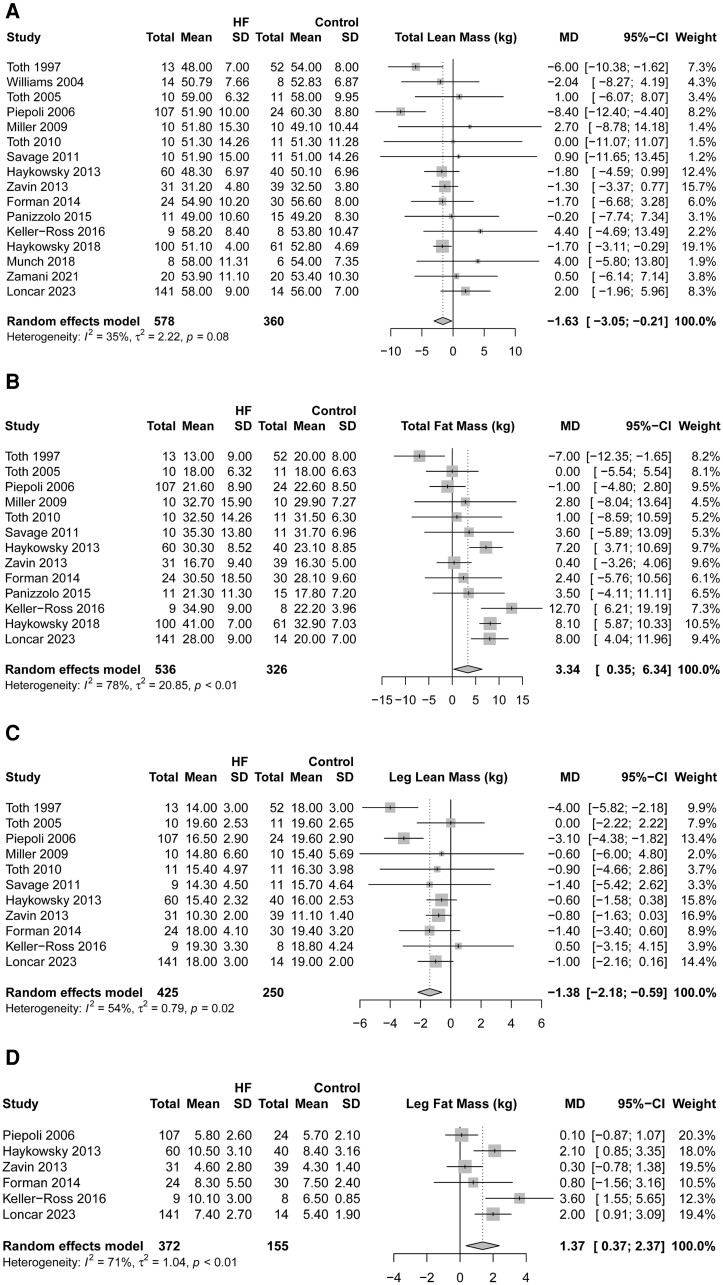
Total body lean mass (kg; *A*), total body fat mass (kg; *B*), leg lean mass (kg; *C*), and leg fat mass (kg; *D*) in patients with heart failure and controls.

**Figure 4:**
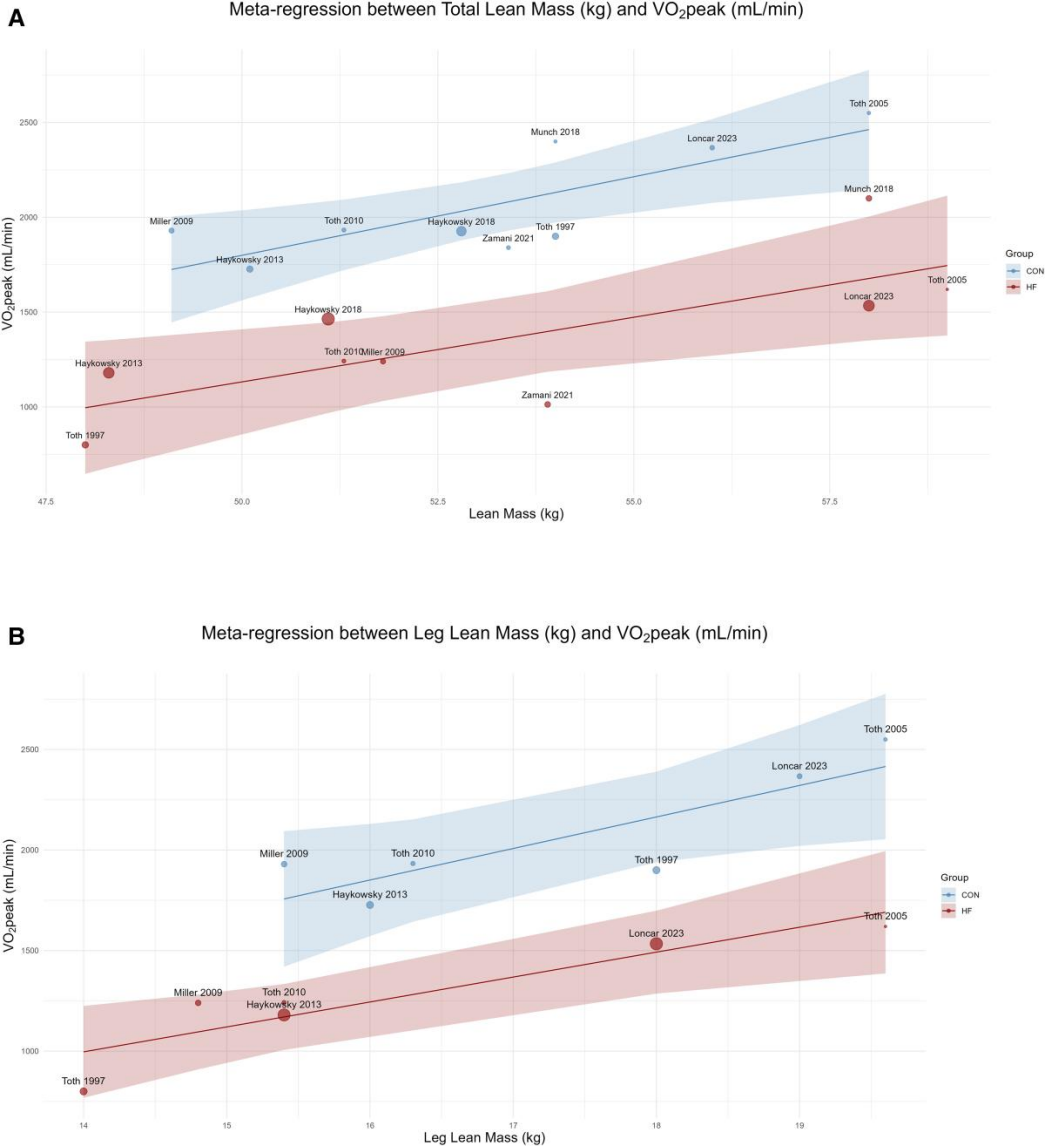
A meta-regression analysis of mean values reported by each study for total body lean mass (kg) and absolute peak oxygen uptake (mL/min; *A*) and leg lean mass (kg) and absolute peak oxygen uptake (mL/min; *B*) in patients with heart failure and controls

Total body lean mass (WMD: −1.63 kg, 95% CI: −3.05 to −0.21 kg, *I*^2^ = 35%, *n* = 938) and leg lean mass (WMD: −1.38 kg, 95% CI: −2.18 to −0.59 kg, *I*^2^ = 54%, *n* = 675) were significantly lower (*Figure 3A* and *C*), while total body fat mass (WMD: 3.34 kg, 95% CI: 0.35–6.34 kg, *I*^2^ = 78%, *n* = 862) and leg fat mass (WMD: 1.37 kg, 95% CI: 0.37–2.37 kg, *I*^2^ = %, *n* = 527) were significantly elevated in HF compared with CON (*Figure 3B* and *D*). Thigh skeletal muscle cross-sectional area (WMD: −10.88 cm^2^, 95% CI: −21.40 to −0.37 cm^2^, *I*^2^ = 64%, *n* = 268) was also significantly reduced in HF relative to CON (see Supplementary material online, *Figure S1*). Furthermore, the absolute VO_2_peak (mL/min) was significantly associated with total body lean mass (HF: β = 68.52, *P* = 0.004, 95% CI: 22.34–114.70; CON: β = 76.17, *P* = 0.001, 95% CI: 29.31–123.03), leg lean mass (HF: β = 145.34, *P* = 0.001, 95% CI: 89.88–200.79; CON: β = 146.49, *P* = 0.008, 95% CI: 38.65–254.34) (*Figure 4A* and *B*), and thigh skeletal muscle cross-sectional area (HF: β = 8.21, *P* < 0.001, 95% CI: 3.83–12.60; CON: β = 27.96, *P* < 0.0001, 95% CI: 15.16–40.75). However, no association was found between VO_2_peak and fat mass (see Supplementary material online, *Table S1*).

### Skeletal muscle morphology

**Figure 5:**
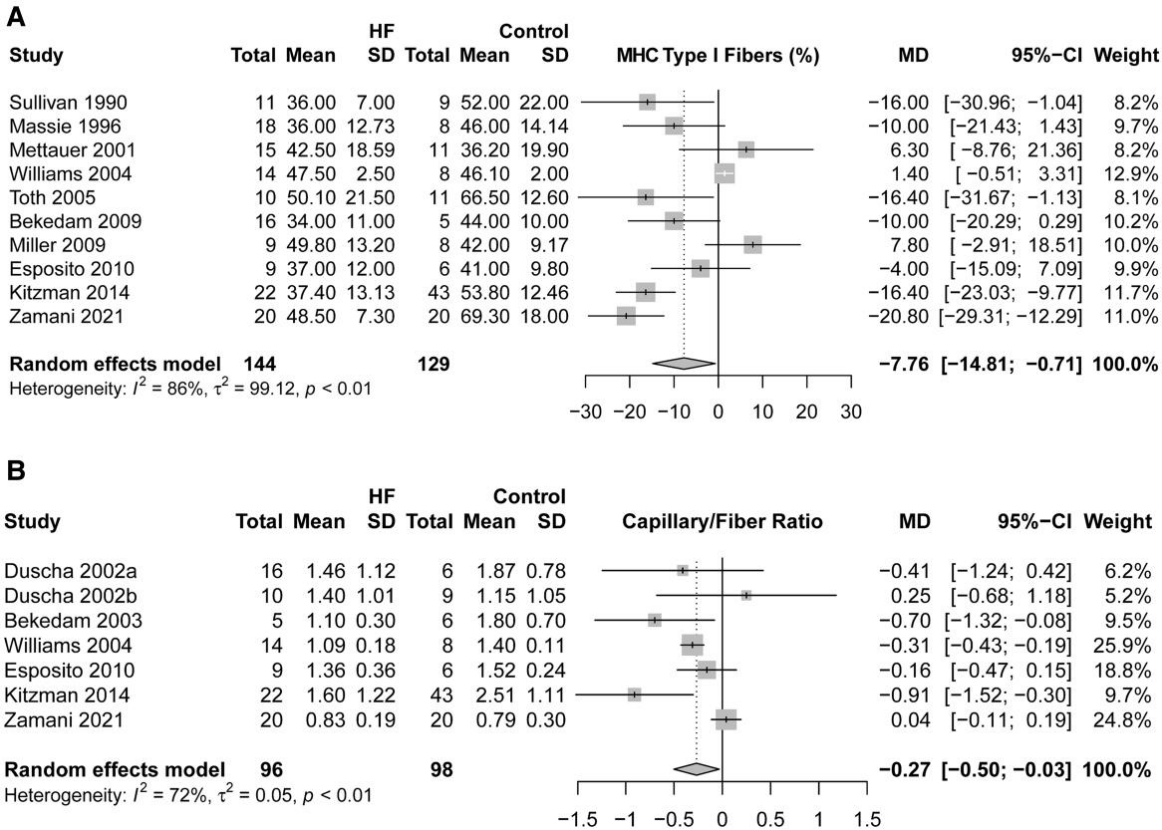
Percentage of myosin heavy chain Type I muscle fibres (%; *A*) and skeletal muscle capillary-to-fibre ratio (*B*) in patients with heart failure and controls.

Compared with CON, HF displayed a significantly lower percentage of Type I muscle fibres (WMD: −7.76%, 95% CI: −14.81 to −0.71%, *I*^2^ = 86%, *n* = 273) (*Figure 5A*). No significant difference was found between groups for Type IIa fibres (WMD: −2.74%, 95% CI: −10.59 to 5.11%, *I*^2^ = 79%, *n* = 172), while the percentage of Type IIx fibres was significantly higher in HF compared with that in CON (WMD: 7.72%, 95% CI: 1.52–13.91, *I*^2^ = 67%, *n* = 126) (see Supplementary material online, *Figure S2*). Additionally, the capillary-to-fibre ratio was markedly lower in HF than in CON (WMD: −0.27, 95% CI: −0.50 to −0.03, *I*^2^ = 72%, *n* = 194) (*Figure 5B*) and also significantly associated with absolute VO_2_peak in HF (HF: β = 584.21, *P* = 0.003, 95% CI: 60.92–394.85; CON: β = −100.86, *P* = 0.501, 95% CI: −394.85 to 193.14). There was no association between the percentage of muscle fibre types and absolute VO_2_peak (see Supplementary material online, *Table S1*).

### Study quality and risk of bias

The risk of bias of the included studies quantified by the AXIS tool was 13.5 points, indicating a moderate risk of bias. The main weaknesses of the included studies were the lack of sample size justification. Detailed AXIS scoring for each study is given in the supplementary material (see Supplementary material online, *Table S2*).

## Discussion

The following are the major new findings of this systematic review and meta-analysis: (i) individuals with HF exhibit a significant and marked reduction in VO_2_peak (∼10 mL/min/kg) compared with CON, and across all studies, the patients with HF displayed an average VO_2_peak of 16 mL/kg/min, which is well below the 18 mL/kg/min VO_2_ threshold required for full and independent living;^32^ (ii) total body and leg lean mass as well as thigh skeletal muscle area are significantly lower in patients with HF vs. CON and this shares a positive relationship to absolute VO_2_peak; and (iii) *m. Vastus lateralis* Type I (oxidative) fibres and capillary-to-fibre ratio were significantly lower in patients with HF vs. CON and capillary-to-fibre ratio was positively associated with absolute VO_2_peak in HF.

### Body composition and exercise intolerance in heart failure

Our findings in the current novel meta-analysis illustrate that muscle atrophy is a major component of the HF syndrome. Specifically, we demonstrate that individuals with HF have reduced total and lower body lean mass and skeletal muscle area relative to CON. Moreover, our analysis also illustrates, across several influential studies, the repercussion of reduced lean mass for exercise intolerance in HF by showing a significant association between total body and leg lean mass with absolute VO_2_peak. These findings align with studies in healthy CON that report a linear relationship between VO_2_peak and lean mass, but not with total body mass.^33,34^ Notably, our findings implicate that leg mass plays a critical role in limiting exercise tolerance as each kilogram increase in lean mass is associated with a concomitant increase in absolute VO_2_peak of about 145 mL/min. Indeed, with a rise in lean mass, the amount of (aerobic) metabolically active tissue presumably increases and thereby the total capacity to utilize the delivered O_2_ for oxidative phosphorylation-dependent generation of adenosine triphosphate for muscle contraction is enhanced.^35^

These findings support the results from a meta-analysis of >1700 patients with HF showing a relatively high prevalence of sarcopenia (34%, 95% CI: 22–47%),^36^ and it is also consistent with several reports showing that individuals with HF who have sarcopenia and/or decreased lean mass have decreased VO_2_peak, reduced physical performance, and lower QoL.^37,38^ Taken together, these findings highlight the importance of targeting skeletal muscle (an important component of lean mass) as a strategy to improve HF-related disability and clinical outcomes, particularly among patients with HF with additional risk factors for sarcopenia (i.e. older patients with HF).^39^ The mechanisms driving the increased muscle atrophy may be related to features inherent to HF such as deconditioning, increased neurohumoral activation, disruption of insulin-like growth factor 1 signalling, pathological systemic and local immune responses, oxidative stress, alterations in protein signalling, and chronic inflammation.^40^ Chronic inflammation is particularly facilitated by fat mass, as adipose tissue promotes the secretion of proinflammatory cytokines such as tumor necrosis factor-alpha and interleukin-6.^41^ These cytokines accelerate the breakdown of muscle proteins and inhibit protein synthesis in muscles, thereby contributing to muscle atrophy. In contrast, anti-inflammatory cytokines such as interleukin-10, cytokine inhibitors such as interleukin-1 receptor antagonist, so-called myokines (which are synthesized and released by muscle tissue during muscle contraction), counteract chronic inflammation.^42,43^

Our results demonstrated that total body and leg fat were 3.3 and 1.4 kg higher, respectively, in HF vs. CON, highlighting not only a transition towards sarcopenia but also a sarcopenic obesity phenotype, which is associated with a poorer prognosis and increased functional limitations.^44^ Nevertheless, our meta-regression analysis revealed no significant association between fat mass and VO_2_peak. This finding may partly be due to collinearity between fat mass and lean mass,^45^ where heavier individuals with HF may also have higher lean mass (and therefore greater absolute VO_2_peak) due to the increased mechanical loading from greater fat and total body mass.^38^ While increased fat mass may not directly reduce VO_2_peak in absolute terms, the metabolic inefficiencies associated with higher fat mass mean that even basic limb movements against gravity require significantly more O_2_ and a larger proportion of an individual’s VO_2_peak. This highlights the importance of interventions targeting increases in muscle and decreases in fat mass, such as combined dietary (e.g. caloric restriction + increased dietary protein intake) and resistance-based exercise interventions.^46^ This may be particularly important to maintain optimal skeletal muscle health and physical function when patients are undergoing medically induced weight loss with bariatric surgery or medications such as glucagon-like peptide-1 agonists, which have the potential to cause marked reductions in lean body mass in addition to their effects on fat mass.^47^

### Skeletal muscle morphology and exercise intolerance in heart failure

In addition to the quantity of lean mass, skeletal muscle quality is also seen as a key factor in exercise intolerance in HF.^24^ Indeed, our meta-analysis indicates that the composition of skeletal muscle fibres in individuals with HF differs from CON. Specifically, the proportion of highly oxidative Type I muscle fibres is reduced by 7.8%, while the proportion of Type IIx (glycolytic) fibres is increased by 7.7% in HF compared with CON. The proportion of oxidative-glycolytic Type IIa fibres also tended to be slightly lower (2.7% lower) in HF. Type I fibres have a high oxidative capacity compared with Type II fibres, especially Type IIx fibres, owing to differences in mitochondrial content, oxidative enzyme activity, and capillarity.^48^ Accordingly, a lower per cent of oxidative fibres would be expected to contribute to decreased VO_2_peak, aerobic performance, and fatigue resistance.^49^ Indeed, Bekedam *et al*.^11^ noted that the cross-sectional area of highly oxidative fibres is relatively small compared with low oxidative fibres, indicating an inverse relationship between fibre cross-sectional area and VO_2_peak.

Additionally, the capillarization of different fibre types is a major determinant of VO_2_peak, which may be impaired in HF.^25,50^ Indeed, the results of our analysis indicate that individuals with HF have a lower capillary-to-fibre ratio and a significant association with VO_2_peak in patients with HF. A relatively higher capillary-to-fibre ratio enhances muscle O_2_ diffusive conductance by reducing the diffusion distance from blood to tissue, increasing O_2_ exchange surface area for O_2_ transport from the microvasculature to the mitochondria, and therefore improving peripheral O_2_ extraction and VO_2_peak.^51^ Ingjer *et al*.^52^ demonstrated that capillarization was highest in Type I fibres and lowest in Type IIx fibres, potentially explaining in part why patients with HF had reduced a proportion of Type I fibres and decreased capillarization in our analysis. Altered muscle fibre composition and capillarity in HF will decrease muscle O_2_ diffusive conductance and O_2_ utilization by skeletal muscles.^53^ Interestingly, we did not observe a significant association between mean study values for muscle fibre composition and VO_2_peak. However, this could be owing to the limited number of studies that concurrently examined VO_2_peak and parameters of muscle fibre composition necessary to perform a meta-regression analysis (Type I fibre %, *n* = 7 studies; Type IIa fibre %, *n* = 4 studies, Type IIx fibre %, *n* = 4 studies, capillary-to-fibre ratio, *n* = 3 studies). Additional research to gain a deeper understanding of this complex relationship in individuals with HF is warranted.

### Skeletal muscle function and exercise intolerance in heart failure

Beyond total body and skeletal muscle composition, there are also additional factors that affect skeletal muscle function that could impact exercise tolerance in HF, such as alterations in microstructural, functional, and biochemical muscle factors. Unfortunately, due to the limited number of studies and variability in their methods and results reporting, we could not incorporate these variables into our meta-analysis. However, the following are the general findings from studies assessing these outcomes. Several studies reported a diminished succinate dehydrogenase, citrate synthase, and 3-hydroxyacyl-CoA dehydrogenase in HF group compared with CON, suggesting a decrease in aerobic oxidative enzyme activity of their skeletal muscle (see Supplementary material online, *Table S3*).^5,9,10,13^ Importantly, the elegant study by Mettauer *et al*.^9^ noted that muscular oxidative capacity (*V*_max_) is decreased in HF compared with active CON, with no significant difference between sedentary CON and HF, suggesting that these differences may be mediated by physical activity rather than HF condition itself. Additionally, the authors observed significantly lower mitochondrial creatine kinase levels in HF, indicating compromised mitochondrial energy supply.^9^ This could be linked to the low percentage of Type I fibres, as these fibres exhibit an increased oxidative enzyme activity and a higher mitochondrial density, contributing to enhanced endurance performance and fatigue resistance.^49^

In contrast to the decreased oxidative enzyme activity and capacity observed in HF, data from our included studies also indicate a tendency for glycolytic enzyme content and activity to increase in patients with HF. This increase could be attributed to the higher proportion of Type IIx fibres, which are characterized by their fast-twitch and quick-fatiguing properties that favour reliance on glycolytic metabolism (see Supplementary material online, *Table S3*).^5,13,49^ Given the predisposition of patients with HF to decreased lean body mass and size, elevations in catabolic markers are another mechanism warranting further investigation in the context of HF, muscle wasting, and exercise intolerance. Although only a few studies have measured the expression of catabolic markers in skeletal muscle in patients with HF, the results are controversial. For instance, while Miller *et al*.^17^ reported no significant differences in the expression of catabolic markers between patients with HF compared to CON, while Forman *et al*.^23^ found elevated expression of atrophy-promoting genes (such as forkhead box proteins O1 and O3 and ubiquitin B), some of which were associated with VO_2_peak.

Taken together, the existing scientific literature indicates a reduction in mitochondrial oxidative metabolism, coupled with increased glycolytic metabolism and up-regulation of catabolic gene expression within skeletal muscle in patients with HF. This shift may contribute to the observed decrease in VO_2_peak and functional limitations in patients with HF. However, due to small sample sizes and variability in study results reporting, methodologies, and control groups, further research is necessary to elucidate the complex relationship between skeletal muscle function and exercise intolerance in patients with HF.

### Limitations

The present analysis is limited by the heterogeneity of the included studies. Firstly, there was a considerable heterogeneity in the study populations in terms of varying sample sizes, sex, HF phenotypes, and HF severity. Moreover, potential differences between HF phenotypes (HFrEF and HFpEF) could not be thoroughly assessed due to limited sample representation, as only 33% of the patients were classified as HFpEF. Additionally, the majority of included patients were male, and no sex-specific analysis was performed; therefore, the generalization of these findings to both sexes should be approached carefully. Comorbidities, and particularly diabetes, may also have contributed to the heterogeneity in body composition outcomes. Unfortunately, the presence of major HF comorbidities such as diabetes was only reported in nine of the included studies (33% of included studies). Furthermore, differences in the characteristics of CON participants may also act as a confounding factor. In most studies, the CON and HF groups were explicitly matched for age and sex, and the mean values for body size were generally well matched between groups. However, as demonstrated in Mettauer *et al*.^9^, the differences in mitochondrial oxidative capacity between HF and CON groups were largely mediated by the active, fitter CON participants, with no significant differences seen when patients with HF were compared with sedentary controls. Although, overall 17 of the 27 included studies (63% of included studies) controlled for physical activity of the CON participants by either matching for physical activity levels of the participants with HF or by excluding participants who reported performing regular physical activity. Notably, at an individual study level, VO_2_peak was significantly lower in HF than sedentary CON groups in all of these studies, and total or leg lean mass was also numerically lower in the vast majority, suggesting that differences in physical activity levels of patients with HF and CON participants were unlikely to explain the overall differences in VO_2_peak or body composition (although it may have contributed to heterogeneity). Despite applying the strict inclusion criteria, the inter-study methodological differences for the reported outcomes represent a further limitation. For one, the use of different CPET modes and protocols may affect the measured VO_2_peak values and their relationship with body and skeletal muscle composition outcomes. Secondly, varying techniques were utilized for the analysis of body composition and muscle biopsies, thus limiting the comparability between the studies.

## Conclusions

Individuals with HF display abnormalities in whole-body and skeletal muscle composition including reduced lean mass, oxidative Type I fibres, and capillary-to-fibre ratio that negatively impact VO_2_peak.

## Lead author biography


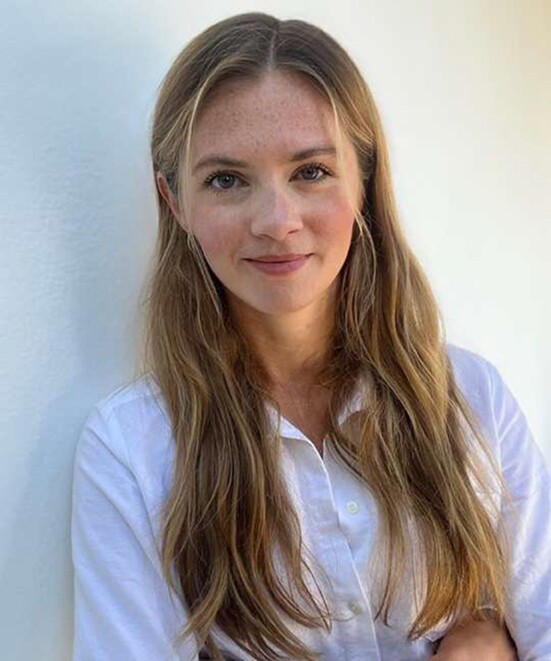


Veronika Schmid is a PhD student at the Department of Preventive Sports Medicine and Sports Cardiology, School of Medicine and Health, Technical University of Munich, in Germany. She holds a master’s degree in Sports Science. Her primary research focuses on heart failure and the role of skeletal muscle in the ageing population. For further information, she can be contacted at veronika.schmid@mri.tum.de.

## Supplementary Material

oeae082_Supplementary_Data

## Data Availability

Not applicable.
