# Supplementary material for: Impact of whole-body and skeletal muscle composition on peak oxygen uptake in heart failure: a systematic review and meta-analysis
Source: Eur Heart J Open. 2024 Sep 26;4(5):oeae082. doi: 10.1093/ehjopen/oeae082 (PMC11505452; doi:10.1093/ehjopen/oeae082)
Supplement: oeae082_Supplementary_Data [file oeae082_supplementary_data.docx]

**Supplemental Materials**

**Title: Impact of whole body and skeletal muscle composition on peak oxygen uptake in heart failure: A Systematic Review and Meta-Analysis**

**Authors**: Veronika Schmid^1,2^, Stephen J. Foulkes^2,3^, Devyn Walesiak^2^, Jing Wang^4^, Corey R. Tomczak^5^, Wesley J. Tucker^6^, Siddhartha S. Angadi^7^, Martin Halle^1^, Mark J. Haykowsky^2^

Affiliations:

^1^ Technical University of Munich, School of Medicine and Health, Department for Preventive Sports Medicine and Sports Cardiology, TUM University Hospital.

^2^ Integrated Cardiovascular Exercise Physiology and Rehabilitation Lab, Faculty of Nursing, College of Health Science, University of Alberta, Edmonton, Alberta, Canada.

^3^ Heart, Exercise and Research Trials Lab, St Vincent’s Institute of Medical Research, Fitzroy, Victoria, Australia.

^4^ Division of Public Health, School of Medicine, University of Utah, Salt Lake City, Utah, USA.

^5^ College of Kinesiology, University of Saskatchewan, Saskatoon Saskatchewan, Canada.

^6^ Department of Nutrition & Food Sciences, Texas Woman’s University, Houston, Texas, USA.

^7^ Department of Kinesiology, University of Virginia, Charlottesville, USA.

^8^ DZHK (German Center for Cardiovascular Research), Partner Site Munich Heart Alliance, Munich, Bavaria, Germany.

**Supplement Figure 1.** Thigh skeletal muscle cross sectional area (CSA, cm^2^) in heart failure (HF) and controls (CON).





**Supplement Figure 2.** Percentage of myosin heavy chain (MHC) type I IIa (%; Panel A) and IIx muscle fibers (%; Panel B) in HF and CON.

**

**

**Supplement Table 1.** Meta-regression analyses with VO_2_peak (mL/min) in HF and CON.

| Factors | k | Estimate | Standard error | Z-value | 95%-CI | | *p-value* |
| --- | --- | --- | --- | --- | --- | --- | --- |
| *Total lean mass (kg)* | | | | | | | |
| HF | 9 | 68.52 | 23.56 | 2.91 | 22.34 to | 114.70 | *0.004* |
| CON | 9 | 76.17 | 23.91 | 3.19 | 29.31 to | 123.03 | *0.001* |
| *Total fat mass (kg)* | | | | | | | |
| HF | 7 | 14.97 | 22.190 | 1.36 | -6.41 to | 35.14 | *0.175* |
| CON | 7 | -21.96 | 17.54 | -1.25 | -5.63 to | 12.43 | *0.211* |
| *Leg lean mass (kg)* | | | | | | | |
| HF | 6 | 145.34 | 28.30 | 5.14 | 89.88 to | 200.79 | *<.0001* |
| CON | 6 | 146.49 | 55.02 | 2.66 | 38.65 to | 254.34 | *0.008* |
| *Thigh skeletal muscle area (cm2)* | | | | | | | |
| HF | 4 | 8.21 | 2.24 | 3.67 | 3.83 to | 12.60 | *<0.001* |
| CON | 4 | 27.96 | 6.53 | 4.28 | 15.16 to | 40.75 | *<.0001* |
| *Fiber type I (%)* | | | | | | | |
| HF | 7 | -15.16 | 14.81 | -1.02 | -44.19 to | 13.88 | *0.306* |
| CON | 7 | -3.14 | 10.58 | -0.30 | -23.89 to | 17.60 | *0.767* |
| *Fiber type IIa (%)* | | | | | | | |
| HF | 4 | 24.68 | 21.41 | 1.15 | -17.29 to | 66.65 | *0.249* |
| CON | 4 | -15.48 | 54.24 | -0.29 | -121.78 to | 90.83 | *0.775* |
| *Fiber type IIx (%)* | | | | | | | |
| HF | 4 | -12.97 | 14.14 | -0.92 | -40.68 to | 14.74 | 0.359 |
| CON | 4 | 3.05 | 19.02 | 0.16 | -34.22 to | 40.33 | 0.873 |
| *Capillary/Fiber ratio* | | | | | | | |
| HF | 3 | 584.21 | 266.99 | 2.19 | 60.92 to | 1107.50 | 0.029 |
| CON | 3 | -100.86 | 150.00 | -0.67 | -394.85 to | 193.14 | 0.501 |

*CI= confidence interval; CON= controls; HF= Heart failure; k= number of studies*

**Supplement Table 2.** Results of study quality assessment using the Axis Tool

|  | Sullivan, 1990 | Massie, 1996 | Harrington, 1997 | Toth, 1997 | Mettauer, 2010 | Duscha, 2002 | Bekedam, 2003 | Schulze, 2004 | Williams, 2004 | Toth, 2005 | Piepoli, 2006 | Bekedam, 2009 | Miller, 2009 | Esposito, 2010 | Toth, 2010 | Savaga, 2011 | Haykowsky, 2013 | Zavin, 2013 | Forman,  2014 | Haykowsky, 2014 | Kitzman, 2014 | Panizzolo, 2015 | Keller-Ross, 2016 | Haykowsky, 2018 | Munch, 2018 | Zamani, 2021 | Loncar, 2023 |  |
| --- | --- | --- | --- | --- | --- | --- | --- | --- | --- | --- | --- | --- | --- | --- | --- | --- | --- | --- | --- | --- | --- | --- | --- | --- | --- | --- | --- | --- |
| **1. Were the aims/objectives of the study clear?** | 1 | 1 | 1 | 1 | 1 | 1 | 1 | 1 | 1 | 1 | 1 | 1 | 1 | 1 | 1 | 1 | 1 | 1 | 1 | 1 | 1 | 1 | 1 | 1 | 1 | 1 | 1 |  |
| **2. Was the study design appropriate for the stated aim(s)?** | 1 | 1 | 1 | 1 | 1 | 1 | 1 | 1 | 1 | 1 | 1 | 1 | 1 | 1 | 1 | 1 | 1 | 1 | 1 | 1 | 1 | 1 | 1 | 1 | 1 | 1 | 1 |  |
| **3. Was the sample size justified?** | 0 | 0 | 0 | 0 | 0 | 0 | 0 | 0 | 0 | 0 | 0 | 0 | 0 | 0 | 0 | 0 | 0 | 0 | 0 | 0 | 0 | 0 | 0 | 0 | 0 | 0 | 0 |  |
| **4. Was the target/reference population clearly defined?  (Is it clear who the research was about?** | 1 | 1 | 1 | 1 | 1 | 1 | 1 | 1 | 1 | 1 | 1 | 1 | 1 | 1 | 1 | 1 | 1 | 1 | 1 | 1 | 1 | 1 | 1 | 1 | 1 | 1 | 1 |  |
| **5. Was the sample frame taken from an appropriate population base so that it closely represented the target/reference population under investigation?** | 0 | 1 | 0 | 1 | 0 | 0 | 1 | 0 | 0 | 1 | 1 | 1 | 0 | 0 | 1 | 0 | 0 | 0 | 1 | 0 | 0 | 0 | 1 | 0 | 1 | 0 | 1 |  |
| **6. Was the selection process likely to select subjects/participants  that were representative of the target/reference population under investigation?** | 0 | 1 | 1 | 1 | 0 | 1 | 0 | 1 | 0 | 0 | 1 | 0 | 0 | 0 | 0 | 1 | 1 | 1 | 1 | 1 | 1 | 1 | 1 | 1 | 1 | 1 | 1 |  |
| **7. Were measures undertaken to address and categorize non-responders?** | 0 | 0 | 0 | 0 | 0 | 0 | 0 | 0 | 0 | 0 | 0 | 0 | 0 | 0 | 0 | 0 | 0 | 0 | 0 | 0 | 0 | 0 | 0 | 0 | 0 | 0 | 0 |  |
| **8. Were the risk factor and outcome variables measured appropriate to the aims of the study?** | 1 | 1 | 1 | 1 | 1 | 1 | 1 | 1 | 1 | 1 | 1 | 1 | 1 | 1 | 1 | 1 | 1 | 1 | 1 | 1 | 1 | 1 | 1 | 1 | 1 | 1 | 1 |  |
| **9. Were the risk factor and outcome variables measured correctly using instruments/ measurements that had been trialled, piloted or published previously?** | 1 | 1 | 1 | 1 | 1 | 1 | 1 | 1 | 1 | 1 | 1 | 1 | 1 | 1 | 1 | 1 | 1 | 1 | 1 | 1 | 1 | 1 | 1 | 1 | 1 | 1 | 1 |  |
| **10. Is it clear what was  to determined statistical significance and/or precision estimates? (e.g. P values CIs)** | 1 | 0 | 1 | 0 | 1 | 1 | 0 | 1 | 1 | 0 | 1 | 1 | 0 | 1 | 0 | 0 | 1 | 1 | 1 | 1 | 1 | 1 | 1 | 1 | 1 | 1 | 1 |  |

**Supplement Table 3.** Markers of skeletal muscle mitochondrial function, enzyme activity and enzyme content in patients with HF versus non-HF controls.

| **Author,**  **Year** | **Phospho-fructokinase** | **Creatine kinase** | **Phosphory-lase** | **Lactate dehydrogenase** | **Succinate dehydrogenase** | **Hexo-kinase** | **Citrate synthase** | **3-Hydroxyacyl-CoA-dehydrogenase** | **Vmax** |
| --- | --- | --- | --- | --- | --- | --- | --- | --- | --- |
| *Sullivan,*  *1990* | ↔ | ↔ | ↔ | ↔ | ↓ |  | ↓ | ↔ |  |
| *Mettauer,*  *2001* |  | ↓ ↔ *^b^* |  |  |  |  |  |  | ↔ ↓ *^b^* |
| *Duscha,*  *2002* |  |  |  |  |  |  |  | ↓ ↔ *^a^* |  |
| *Bekedam,*  *2003* |  |  |  |  | ↓ |  |  |  |  |
| *Williams,*  *2004* | ↔ |  |  | ↔ |  |  | ↔ | ↔ |  |
| *Bekedam,*  *2009* |  |  |  |  | ↓ |  |  |  |  |
| *Miller,*  *2009* |  |  |  |  |  |  |  |  |  |
| *Esposito,*  *2010* |  |  |  |  |  |  |  |  |  |
| *Zamani,*  *2021* |  |  |  |  |  |  | ↔ |  |  |

*Vmax= muscular oxidative capacity,
 ^a^ male & female, ^b^ vs. sedentary & active,
 ↓= significant lower values in HF vs. CON, ↔ = No significant difference in HF vs. CON*
